# Expression of concern: Microbiome-based hypothesis on Ivermectin's mechanism in COVID-19: Ivermectin feeds bifidobacteria to boost immunity

**DOI:** 10.3389/fmicb.2022.1128469

**Published:** 2023-01-05

**Authors:** Frontiers Editorial Office

With this notice, Frontiers states its awareness of the serious concerns raised regarding this article, which are now under investigation. The situation will be updated as soon as the investigation is complete.

